# Epithelial architecture and signaling activity in the adult human esophagus

**DOI:** 10.3389/fcell.2025.1632255

**Published:** 2025-07-16

**Authors:** David Grommisch, Evelien Eenjes, Maeve L. Troost, Maria Genander

**Affiliations:** ^1^ Max Planck Institute of Molecular Cell Biology and Genetics (MPI-CBG), Dresden, Germany; ^2^ Department of Cell and Molecular Biology, Karolinska Institutet, Stockholm, Sweden

**Keywords:** esophagus, epithelium, signaling, progenitor cells, culture systems, organoid, adult stem cell

## Abstract

Barrier epithelia function to shield the inside of our bodies from external stressors and pathogens. The esophageal epithelium is no exception, providing protection while at the same time transporting food to the stomach. Although many epithelial tissues are comparable between humans and mice, the human esophageal epithelium displays unique features in both progenitor cell organization and tissue architecture compared to the mouse. These differences have limited our understanding of the adult human esophagus, hindering the development of therapeutic strategies targeting human esophageal disease. Herein, we contrast the esophageal epithelial architecture and progenitor cell populations in mice and humans and discuss the role of a tentative human-specific progenitor cell population located in the submucosal gland ducts. Furthermore, we review current models available to study the human esophageal epithelium, focusing predominantly on adult primary organoids and epithelioids as well as the generation of human developmental esophageal epithelial cells from induced pluripotent stem cells. Finally, we discuss signaling activity implicated in maintaining normal human epithelial homeostasis, and how these pathways contribute to disease development. We aim to provide a comprehensive outlook on our current understanding of the human esophageal epithelium, while simultaneously highlighting unanswered questions in esophageal epithelial maintenance.

## The esophageal epithelium

Maintaining a functioning epithelial barrier is required for human survival, combining protection from the outside world with tissue-specific functions. The esophageal epithelium ensures continued integrity by endlessly generating new epithelial cells which undergo a stereotyped and coordinated process of differentiation, replacing the entire epithelium within days. Rapid tissue turnover requires the proliferation of esophageal progenitor cells, residing strategically within the epithelium to be able to respond swiftly to changes in the microenvironment. Failure to regulate progenitor cell behavior leads to esophageal dysfunction, often manifested with barrier defects and hyperplasia.

In contrast to other epithelial tissues, differences in esophageal tissue architecture are striking when comparing species. Species-specific distinctions may be the result of an evolutionary adaptation to a combination of factors, including the texture of the food and efficiency of the masticatory system, which collectively impact the softness of the food bolus passing through the esophageal tube. Herein, we explore parallels and highlight differences in the adult mouse and human esophageal epithelium, underscoring areas where future research is required to propel development of promising tools and strategies targeting esophageal disease.

## Comparative description of the mouse and human esophageal architecture

**FIGURE 1:**
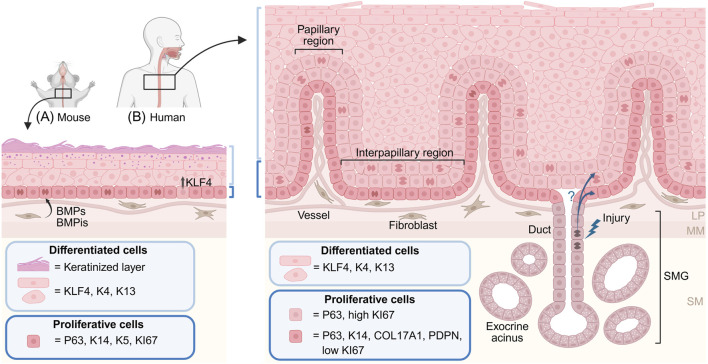
Mouse and human esophageal epithelial architecture. Illustration of the mouse **(A)** and human **(B)** esophageal squamous epithelium. The mouse epithelium **(A)** is maintained by a single layer of basal proliferating progenitor cells expressing P63, K14 and K5. Upregulation of KLF4 in basal cells initiates epithelial differentiation and delamination from the basal layer. Suprabasal cells undergo terminal differentiation and give rise to a keratinized outer cell layer. Fibroblasts in the lamina propria secrete signaling molecules instructive to basal progenitor cells, including BMP ligands (BMPs) and inhibitors (BMPis). **(B)** The human epithelium is folded into papillary and interpapillary regions. The basal layer, marked by P63, K14, COL17A1 and PDPN, harbors slow cycling progenitor cells, whereas highly proliferative P63-retaining cells are found in the first suprabasal layers. Differentiated human epithelial cells express largely similar markers as the mouse counterparts, but do not undergo keratinization. Instead, the human esophagus contains submucosal glands (SMGs) consisting of exocrine mucus-producing acini connected to the esophageal epithelium through glandular ducts. It has been proposed that duct cells can become activated, and contribute to re-epithelialization, during esophageal injury. LM: lamina propria, MM: muscularis mucosae, SM: submucosa.

The adult mouse esophageal epithelium is a three-to-four cell layer thick squamous keratinized epithelium (Figure 1A), which is gently folded around the tube circumference to enable expansion upon passing of the food-bolus. The epithelial basal layer contains proliferating (KI67^+^) progenitor cells, marked by K14, K5 and P63 (Rosekrans et al., 2015). Once basal layer progenitor cells upregulate KLF4 (McGinn et al., 2021) (Figure 1A), they commit to differentiation and delaminate out from the basal layer to eventually shed off into the esophageal lumen. The continuous proliferation of basal cells is estimated to renew the esophageal epithelium every 3.5 days (Doupe et al., 2012). While the overall mouse esophageal architecture is strikingly similar to the mouse interfollicular epidermis (Piedrafita et al., 2020), the esophageal epithelium lacks epithelial appendages like the hair follicles and sweat glands of the skin. Recent work, however, describes the presence of rare specialized epithelial taste buds in the upper mouse esophagus (Vercauteren Drubbel and Beck, 2023) indicating that the mouse epithelium is more complex than previously thought.

In contrast to the mouse epithelium, the human esophageal epithelium is up to 40 cell layers thick and displays prominent stromal invaginations, which folds the epithelium into structures called papillae (Geboes and Desmet, 1978) (Figure 1B), reminiscent of the patterning of the human epidermis. The human basal layer is marked by K14, P63, COL17A1 and PDPN (Busslinger et al., 2021; Rochman et al., 2022; Rosekrans et al., 2015) and is largely quiescent (Figure 1B). Proliferating P63-positive progenitor cells locate up to the first six suprabasal cell layers, suggesting a more complex tissue organization than what is found in the mouse epithelium. Epithelial cell layers above the proliferating zone express KLF4, K4 and K13, markers associated with lineage commitment and terminal differentiation. Although the keratin expression profile shifts with human esophageal epithelial differentiation, the keratinized cell layer - present in the mouse epithelium - is absent in the human esophagus. Instead, the human esophagus contains submucosal glands (SMGs) (Figure 1B), which safeguard the surface squamous epithelium by secreting mucus (Long and Orlando, 1999). The turnover time of the human esophageal epithelium is estimated to be around 11 days (Pan et al., 2013).

Due to its simple architecture, the adult mouse esophagus presents an ideal system to uncover mechanisms of epithelial development and homeostasis, and has contributed significantly to our understanding of adult epithelial tissue maintenance and fitness (Abby et al., 2023; Doupe et al., 2012; Frede et al., 2016; Piedrafita et al., 2020). However, the increased complexity of the human epithelial architecture limits translational efforts, especially when probing cell- and signaling-mechanisms related to the development of human esophageal disease.

## Differences in behavior and number–comparing esophageal progenitor cell populations

Stem and progenitor cell behavior is dictated by the local environment, or niche (Lane et al., 2014). The mouse esophageal epithelium, being structurally simple, has no obvious anatomical landmarks that would allow discrete progenitor cell - niche cell interactions. Hence, work using strategies to genetically label an unbiased fraction of basal progenitor cells demonstrate that the esophageal epithelium is maintained by a single progenitor cell population, dividing symmetrically twice per week (Doupe et al., 2012). Daughter cells retain progenitor cell properties or commit to lineage differentiation in a stochastic, random, manner (Doupe et al., 2012). Although this model describes the mouse esophageal tissue well, it does not formally exclude the existence of subset(s) of progenitor cells which behave differently from the bulk population.

Despite the lack of clear anatomical epithelial niches, gene expression within the mouse epithelium is not uniform. Irregular epithelial expression of signaling molecules, including *Bmp4*, *Gli1, Igfbp2* and *Igfbp5* suggest that restricted signaling environments could act to diversify behavior in discrete subsets of progenitor cells (Grommisch et al., 2025; Jiang et al., 2015; van Dop et al., 2013). In support of this idea, several studies targeting specific progenitor subpopulations have indicated that heterogeneity within the mouse esophageal progenitor pool exists (Croagh et al., 2007; DeWard et al., 2014; Giroux et al., 2017; Grommisch et al., 2024; Kalabis et al., 2008), reporting differences in *in vivo* basal clone sizes and in *in vitro* organoid-forming potential. In addition, recent single-cell profiling reports that local non-epithelial niche cell architectures differ in the upper (proximal) and lower (distal) esophagus (Grommisch et al., 2025), and demonstrates a distinct, distally enriched, epithelial progenitor population (Grommisch et al., 2025; Grommisch et al., 2024). These findings indicate that the regulation of progenitor cell states and behaviors in the mouse esophageal epithelium could be more complex than previously appreciated. The rapid development of *in situ* sequencing technologies will likely shed new light on thus far hidden progenitor niches and local cellular networks, enhancing our understanding of how progenitor cell behavior is restricted and diversified in structurally simple tissues such as the mouse esophageal epithelium.

The nature of the human esophageal epithelial progenitor pool is less characterized. With the availability of high-resolution sequencing data (Busslinger et al., 2021; Chen et al., 2021; Ding et al., 2024; He et al., 2020; Madissoon et al., 2019; Rochman et al., 2022), we have gained in-depth information about the transcriptional states defining basal and suprabasal cell populations in the human esophagus, differences which may correspond to distinct cellular behaviors. Translating transcriptional cell states into lineage hierarchies and cell behaviors has however proven difficult. Attempts to identify and functionally compare potential progenitor cell populations in the human epithelium include fractionation based on cell surface markers. Separating basal-to-suprabasal populations using cell surface markers ITGA6 or PDPN (Jeong et al., 2016; Rochman et al., 2022) demonstrates high clonogenic capacity in the basal, normally slow cycling, cell population. Taken together, this suggests that basal cells, although largely quiescent *in vivo*, represent a progenitor population that efficiently forms 2D colonies *in vitro*. It is possible that proliferating suprabasal cells represent a more transient cell state already *en route* towards differentiation, as indicated by their low colony forming ability. This would suggest that the human esophageal epithelium is maintained by two distinct progenitor cell populations which can be identified based on their location (basal vs. suprabasal) and proliferation kinetics (slow vs. actively cycling). How they are related hierarchically, and how long each population resides in the epithelium, is still to be addressed.

In addition, basal cells located to folded papillary regions find themselves in a different local environment compared to basal cells in the interpapillary regions (Figure 1B), potentially affecting their progenitor cell potential and behavior. In the human epidermis, topographical location affects basal cell clonogenicity (Jones et al., 1995), suggesting that the same could be true in the esophagus. However, attempts to subset the basal layer into ITGB1^high^ papillary and ITGB1^low^ interpapillary cell populations and compare *in vitro* clonogenicity were largely inconclusive (Seery and Watt, 2000). Available single-cell transcriptional data could be explored to investigate if additional heterogeneity within the basal layer, indicative of distinct papillary-interpapillary cell states, exist.

## Human esophageal glands – adding tissue complexity

To protect the non-keratinized human epithelium from damage, submucosal glands (SMGs) are scattered throughout the human esophagus (Figure 1B). Each SMG is composed of multiple exocrine acini which produce mainly mucus, but also bicarbonates and EGF (Epidermal Growth Factor), to facilitate food bolus transport, neutralize stomach acids and promote epithelial regeneration (von Furstenberg et al., 2017). Each acinus is drained by a small duct, which coalesce into one large duct transversing the submucosa and epithelium to allow the SMGs to release their content into the esophageal lumen. Duct cells near the SMG are cuboidal in shape, but transition into a squamous appearance as the duct approaches the lumen of the esophageal tube (Garman, 2017; Garman et al., 2015). Proliferation in SMGs and associated ducts is low during homeostasis but increases in pig and canine models of epithelial damage, including experimentally induced acid reflux (Gillen et al., 1988; Li et al., 1994; Van Nieuwenhove and Willems, 1998; von Furstenberg et al., 2017). Data from animal models also suggest that submucosal glands and/or their connecting ducts may contribute to esophageal epithelial repair upon injury (Gillen et al., 1988; Kruger et al., 2017; Owen et al., 2018), paralleling findings in mice describing activation and contribution of sweat gland duct progenitor cells to the re-epithelialization of the epidermis (Lu et al., 2012). Duct progenitor cells could thus represent a so-far uncharacterized reservoir of latent progenitor cells which can be activated and recruited to contribute to the healing of the squamous esophageal epithelium.

SMG duct cells have attracted attention as a potential cell-of-origin in Barrett’s metaplasia of the esophagus (BE), a condition where the distal part of the human squamous epithelium is replaced by a mosaic of columnar gastric and intestinal cell types, which can progress to dysplasia and adenocarcinoma formation (Jung et al., 2011). The development of BE is linked to repetitive stomach acid reflux and thus initiates at the gastro-esophageal junction. Due to differences in mouse and human gastro-esophageal junctional anatomy (Caron et al., 2015), the cellular origin of BE has proven challenging to formally verify in humans. Although mice, lacking SMGs, can develop metaplastic lesions upon injury (Vercauteren Drubbel et al., 2021), clonal genetic analysis has recorded matching somatic mutation profiles in manually dissected human SMG duct cells and neighboring Barrett’s glandular tissue, indicating a common cellular origin (Leedham et al., 2008). Single cell transcriptional profiling also concluded that the SMG duct cells are transcriptionally closer to cells found in BE, when compared to cells normally located in the intestine or gastric cardia (Owen et al., 2018). However, more recent in-depth chromatin analysis excluded the SMG duct and instead pinpointed the gastric cardia as the cell-of-origin in BE (Nowicki-Osuch et al., 2021). Considering the possibility that multiple progenitor populations could drive the histogenesis of BE would perhaps reconcile these seemingly opposing findings.

## Recapitulate features of the human esophageal epithelium *in vitro*

Much of our mechanistic understanding of how the human esophageal epithelium is maintained is extrapolated from work in the mouse. Efforts identifying and functionally characterizing key signaling pathways in the adult human epithelium come from mapping somatic disease-causing mutations (Lin et al., 2018; Liu et al., 2017; Martincorena et al., 2018; Zhang et al., 2015) and establishing culture conditions permissive for maintenance and differentiation of esophageal epithelial cells (Kalabis et al., 2012; Laczko et al., 2017; Milne et al., 2024; Mou et al., 2016; Sachdeva et al., 2021; Urano et al., 2025). In addition, reactivation of important developmental pathways is not uncommon in esophageal disease (Vercauteren Drubbel et al., 2021), some of which have successfully and sequentially been exploited to generate esophageal epithelial basal-like cells from human pluripotent stem cells (hPSCs) (Bailey et al., 2019; Ferrer-Torres et al., 2022; Nakagawa et al., 2020; Trisno et al., 2018). Despite these recent advances, a robust, reproducible and readily available esophageal epithelial cell model is still lacking.

### Exploiting adult primary esophageal epithelial cells

Organoids have proven a powerful tool to understand mechanisms of homeostasis and regeneration in several epithelia. Several organoid culturing conditions for mouse esophageal basal cells are established (DeWard et al., 2014; Kasagi et al., 2018) producing organoids that reflect the mouse esophageal epithelial tissue architecture well. Culturing of mouse organoids requires the presence of EGF in combination with either Ca^2+^ (Kasagi et al., 2018) or Noggin (BMP inhibitor) and R-spondin (a WNT signaling agonist) (DeWard et al., 2014). Although no upper limitation is reported on passaging of mouse organoids, passaging involves regular dissociation and replating of single basal cells, shortening the temporal window available for analysis of individual cell behavior. In this regard, organoids are not an ideal model for understanding basal cell states during esophageal homeostasis but rather represents a versatile, high throughput, system for probing cell and signaling cues governing epithelial cell states, be it proximal vs. distal epithelial basal cell identities (Grommisch et al., 2025) or disease initiating mechanisms (Kasagi et al., 2018).

Complementing the use of mouse organoids are epithelioids (Herms et al., 2024), formed by expanding primary esophageal epithelial basal progenitor cells which initiate differentiation when reaching confluency. Differentiation correlates to stratification, generating a 3D multilayered epithelial sheet which reaches homeostasis and can be maintained long-term in culture without passaging. Esophageal epithelioids derived from primary human esophageal epithelium are reported (Herms et al., 2024), but do not recapitulate the complexity of the human esophageal epithelial architecture. Nevertheless, epithelioids will likely prove useful in addressing the functional outcome of somatic mutations or mechanisms of squamous-to-columnar transdifferentiation and transformation, processes which require a longer experimental time window than currently provided by organoids.

In contrast to the successful establishment of mouse esophageal organoids, the field has so far failed to maintain adult human esophageal organoids in culture for more than a handful of passages. This limits the use of human esophageal organoids and increases the need for a continuous supply of human resection material. A variety of culture conditions have been reported (see Table 1); however, they are largely similar and fail to maintain organoid-forming capacity over time. Considering that the basal cells in the human epithelium are not rapidly cycling *in vivo*, it is possible that current organoid culture conditions, promoting cell cycle entry and expansion, exhaust this slow cycling, potentially long-lived, progenitor population. Instead, organoid media conditions may favor enrichment of proliferating progenitor cell states, corresponding to the first layers of *in vivo* suprabasal cell states. Activation, and subsequent loss, of the normally slow-cycling progenitor population would thus infer that epithelial cell state heterogeneity is reduced *in vitro*, thereby potentially hampering long-term passaging ability. How to maintain a largely quiescent progenitor pool, while simultaneously promoting organoid expansion remains an interesting challenge to resolve.

**TABLE 1:** Outlining of esophageal primary cell, organoid and hPSC culture conditions.

Culture	Media	Medium supplements	Reference
2D Primary P2	KSFM	BPE, 0.09 mM Ca^2+^	Kaymak and Niess (2024), Andl et al. (2003), Harada et al. (2003), Kalabis et al. (2012) ^a^, Giroux et al. (2017) ^a^
		EGF	
2D Primary P3	DMEM/F12	N2, B27 w/o VitA, NAC, HC, Feeders (3T3-J2i)	Ferrer-Torres et al. (2022)
		Noggin, EGF	
		CHIR99021, A83-01, Y-27632	
2D hPSC P2	F12: IMDM (1:4)	N2, B27, Bovine Albumin, L-Ascorbic acid, MTG	Yang et al. (2025)
		EGF, BMP4,TGFB1	
		IWP2	
Epithelioid Primary	DMEM/F12	FCS, Insulin, Adenine, Cholera Toxin, HC, Apo-trans	Herms et al. (2024)
		EGF	
Organoid Primary P3	KSFM	BPE, 0.6 mM Ca^2+^	Kasagi et al. (2018), Kaymak and Niess (2024)
		EGF	
		Y-27632	
Organoid Primary	DMEM/F12	N2, B27, NAC, Nicotinamide	Giroux et al. (2017)
		EGF, Noggin^b^, RSPO^b^, Wnt3a, Gastrin	
		A83-01, SB202190, Y-27632	
Organoid Primary P3-4	Adv. DMEM/F12	B27, NAC, Nicotinamide	Nakagawa et al. (2025)
		EGF, Noggin, RSPO^b^, FGF2	
		A83-01, PGE2, Forskolin, CHIR99021, Y-27632	
Organoid Primary P6-7	Adv. DMEM/F12	EGF	Busslinger et al. (2021)
		Y-27632	
		A83-01, SB202190, Y-27632 (after plating)	
Organoid hPSC	Adv. DMEM/F12	B27, N2	Trisno et al. (2018)
		EGF, Noggin (first 3 days), FGF10 (first 7 days)	
Organoid hPSC	F12: IMDM (1:4)	B27, N2, Bovine Albumin, L-Ascorbic acid, MTG	Zhang Y. et al. (2018)
		EGF, Noggin, FGF2	
		CHIR99021, SB431542	

Y-27632, Rock inhibitor; A83-01, ALK4/5/7 inhibitor; DMH1, ALK2 inhibitor; SB202190, p38/MAPK; inhibitor; CHIR99021, GSK-3; inhibitor; SB431542, ALK4/5/7 inhibitor; MTG, 1-Thioglycerol; IWP2, WNT; inhibitor; RSPO, R-Spondin; NAC, N-acetyl-L-cystein; HC, hydrocortisone; FCS, fetal calf serum; FSK, forskolin; EGF, epithelial growth factor; FGF, fibroblast growth factors; PGE2, Prostaglandin E2.

^a^ 0.018 mM CaCl_2_.

^b^ Conditioned medium; Passaging (P) indicated when relevant.

Based on the following references (Andl et al., 2003; Busslinger et al., 2021; Ferrer-Torres et al., 2022; Giroux et al., 2017; Harada et al., 2003; Herms et al., 2024; Kalabis et al., 2012; Kasagi et al., 2018; Kaymak and Niess, 2024; Nakagawa et al., 2025; Trisno et al., 2018; Yang et al., 2025; Zhang Y. et al., 2018).

With the emergence of single-cell omics, comparisons between cultured human primary cells and *in vivo* epithelial cell states are now feasible and will likely aid in defining organoid culture conditions enriching for either slow cycling or activated basal cell states, but also in determining the physiological relevance of different *in vitro* systems, thereby guiding further model refinement. While a direct primary-to-organoid human esophageal basal cell state comparison is still outstanding, insights from other epithelial tissues indicate that adult esophageal basal cells could adopt a more immature or embryonic cell state when in culture (Ortiz et al., 2024), potentially analogous to the reactivation of embryonic transcriptional signatures described during epithelial regeneration (Viragova et al., 2024).

### Generating *de novo* human esophageal epithelial cells

Human pluripotent stem cell (hPSC)-derived esophageal epithelial cells represent an additional tool for understanding esophageal homeostasis and disease. Drawing from human development, esophageal epithelial progenitor cells can be specified from the foregut endoderm (Koterazawa et al., 2020; Trisno et al., 2018; Yang et al., 2025; Zhang Y. et al., 2018). Common specification strategies suggest that silencing of WNT and activation of RA (Retinoic Acid) activity, in combination with dual BMP/TGFβ inhibition, is critical for generating immature esophageal progenitor cells. Reactivation of BMP/TGFβ signaling together with EGF is then required for further specification, and expansion, of esophageal basal cells (Yang et al., 2025). These strategies currently do not generate pure esophageal basal cell cultures and therefore rely on continuous cell sorting to isolate basal cells for subsequent functional testing. In addition, hPSC-derived basal cells represent embryonic cell states, which may differ from the adult basal cells that can be isolated from human esophagi. Direct transcriptional comparisons are still missing but would be valuable for understanding subtle differences in cell behavior. Despite these current shortcomings, hPSCs-derived esophageal basal cells represent a potential unlimited source of cells, which will undoubtedly further our understanding of not only esophageal development (Trisno et al., 2018; Zhang Y. et al., 2018), but also homeostasis and disease (Bailey et al., 2019).

## Harnessing tissue function to establish relevant cell models

Here we provide a short overview of the current knowledge of key pathways known to affect either development of esophageal disease, or expansion and maintenance of esophageal epithelial cells in culture. The suboptimal conditions used for primary and hPSC cell models suggest that additional, yet unidentified growth factors or signalling cues may be required to establish a cell platform that accurately captures *in vivo* epithelial progenitor heterogeneity and models adult disease initiation.

### Reactivation of developmental pathways–role of SHH and RA signaling in esophageal disease

Developing epithelial cells states are commonly reactivated during disease and regeneration (Viragova et al., 2024). Signaling pathways such as SHH (Sonic hedgehog) and RA (Retinoic Acid) are active during esophageal development (Zhang et al., 2021; Zhang et al., 2017) and subsequently reactivated in adult human BE (Chang et al., 2007; Vercauteren Drubbel et al., 2021; Yamanaka et al., 2011). SHH and RA both act to rewire squamous epithelial transcriptional cell identities towards columnar metaplastic states (Chang et al., 2007; Vercauteren Drubbel et al., 2021), indicating that repression of SHH and RA signaling activity is required to maintain the identity of the adult esophageal squamous epithelium. Casting a wider web, reactivation of SHH signaling in the adult esophagus is linked to downstream BMP activation (Wang et al., 2010), whereas RA activity is suggested to induce canonical WNT signaling (Mao et al., 2018), thus demonstrating how reactivation of developmental pathways enables hijacking and rewiring of transcriptional networks required for normal esophageal epithelial homeostasis.

### Committing to differentiation–BMP signaling

BMP signaling commonly acts to restrict proliferation and enable differentiation in adult epithelia. The same is true in the mouse esophagus (Rodriguez et al., 2010). BMP ligands are produced by the epithelium itself (Grommisch et al., 2025; Jiang et al., 2015), but also by fibroblasts, preferentially found in the distal esophagus (Grommisch et al., 2025). BMP reporter expression, a proxy for active BMP signaling, is however limited to suprabasal epithelial cell layers (Jiang et al., 2015), indicating that BMP signaling is low in proliferating basal cells, only to be increased as cells commit to delamination and differentiation. In line with this notion, stromal cells produce (in addition to BMP ligands) BMP inhibitors which could serve to repress BMP signaling in the epithelial basal layer (Grommisch et al., 2025; Jiang et al., 2015) (Figure 1A). Regulation and sensing of BMP ligands are thus tightly regulated during adult mouse esophageal homeostasis. The role of BMP signaling in the human esophageal epithelium is not fully explored, but human organoids fail to grow in the absence of the BMP inhibitor Noggin (Zheng et al., 2021), phenocopying mouse organoids and indicating a similar requirement to suppress BMP signaling to promote renewing progenitor cell proliferation.

Reactivation of SHH during the development of BE induces stromal BMP4 production which drives esophageal squamous-to-columnar epithelial transformation *in vitro*, and the appearance of columnar intestinal epithelium in the esophagus *in vivo* (Correia et al., 2023; Mari et al., 2014; Vercauteren Drubbel et al., 2021; Wang et al., 2010; Zhang C. et al., 2018). Interestingly, forced BMP signaling in the mouse esophagus inhibits the normal developmental columnar-to-squamous transition in the distal esophagus (Rodriguez et al., 2010). These data indicate that BMP signaling could have two distinct roles in the adult human epithelium – enabling squamous cell differentiation during homeostasis and, upon reactivation and in combination with additional developmental signaling programs, favoring columnar epithelial cell states.

### On the lookout for non-canonical signatures–repression of WNT

The role of canonical WNT signaling in the esophagus is poorly understood, in striking contrast to the wealth of information describing WNT signaling in other adult epithelia (Clevers et al., 2014). During early human esophageal development, suppression of WNT is required to specify esophageal progenitor cells from the dorsal anterior foregut (Harris-Johnson et al., 2009; Trisno et al., 2018; Woo et al., 2011). In the adult human esophageal epithelium, the role of WNT signaling is unknown, but expression of canonical WNT target genes are low in the mouse epithelium (Grommisch et al., 2024), suggesting again that suppression, rather than activation, of epithelial WNT signaling is required for maintaining esophageal epithelial cell states. To this end, somatic epithelial WNT mutations are not common (Cancer Genome Atlas Research et al., 2017; Lin et al., 2014; Martincorena et al., 2018; Song et al., 2014), and signs of canonical WNT activity are only reported in cases of progressed dysplastic BE (Bian et al., 2000; Moyes et al., 2012; Osterheld et al., 2002), likely associated with the progressive appearance of intestinal columnar, WNT-dependent, cell states. These observations would suggest that canonical WNT signaling is not linked to the development of Barrett’s esophagus *per se*, but rather a driver of subsequent malignant progression.

Despite the lack of evidence for canonical WNT signaling activity during epithelial homeostasis, both the mouse and human epithelium express WNTs and WNT signaling mediators – including non-canonical WNT ligands (Busslinger et al., 2021; Grommisch et al., 2025). While removal of canonical WNT agonist R-spondin is compatible with mouse organoid growth and passaging (Kumar et al., 2024), addition of exogenous non-canonical WNT5a significantly reduces organoid forming capacity (Grommisch et al., 2025). These findings point towards a so-far unexplored role for non-canonical WNT signaling in impacting mouse epithelial maintenance, potentially mirrored by the human epithelium.

### Coupling tumor suppression to basal cell expansion–intriguing Notch signaling

Notch receptors are enriched in human suprabasal proliferating epithelial progenitor cells, whereas Jagged2 and Delta like-1, two Notch-receptor ligands, are found in the slow cycling basal cell layer (Busslinger et al., 2021). Interestingly, in contrast to the reported absence of human somatic WNT mutations (Martincorena et al., 2018), loss-of-function Notch1/3 mutations are common in ageing human esophagus, although underrepresented in human esophageal squamous cell carcinomas (Abby et al., 2023). Elegant work in the mouse epithelium demonstrates how ablation of Notch1 in the adult epithelium drives clonal expansion of Notch1-deficient basal cells during homeostasis but at the same time, impairs tumor formation (Abby et al., 2023), revealing how inactivating Notch1 mutations in human epithelia could couple progenitor cell expansion with tumor suppression. In human organoids, inhibiting Notch signaling results in an accumulation of basal cells, while restricting differentiation (Kasagi et al., 2018; Ohashi et al., 2010). It is possible that a partial or transient suppression of Notch activity in human organoids could act directly on restricting commitment to differentiation in basal cells, thereby enabling the upkeep of slow cycling basal cell states permissive for long-term organoid renewal.

Step-by-step we identify mechanisms of esophageal epithelial maintenance, delineating specific roles of signaling pathways in directing cell states and behaviors. However, our inability to maintain long-lasting human esophageal epithelial cultures clearly demonstrates that our understanding of how the esophageal epithelium is regulated is incomplete. Future integration of 3D culture systems and organoid co-culture approaches may offer promising avenues to more accurately recapitulate the complex epithelial-niche interactions and signaling governing esophageal epithelial homeostasis and pathology.

## Outlook

The last two decades have recorded groundbreaking insights in the cell biological mechanisms regulating tissue development and homeostasis. For reasons unknown, interest in the esophageal epithelium has been comparably limited. We therefore still lack many of the pieces required to complete the puzzle of esophageal tissue maintenance. Ambitious transcriptional, largely descriptive, data is emerging allowing for further in-depth mechanistic studies, both in terms of understanding epithelial progenitor biology, but also detailing cell-to-cell interaction networks and disease-associated transcriptional profiles. In parallel, available human cell models required to test new hypothesis are continuously improving, allowing researchers to ask more targeted questions. The recent hPSC-derived esophageal mesenchymal cells (Han et al., 2020; Kishimoto et al., 2022) are but one example of a valuable tool for understanding human esophageal development and disease. We hope that this review article will provide inspiration to future work dedicated to advancing our understanding of this intriguing epithelial tube.
